# Inhibition of SHIP2 in CD2AP-deficient podocytes ameliorates reactive oxygen species generation but aggravates apoptosis

**DOI:** 10.1038/s41598-017-10512-w

**Published:** 2017-09-06

**Authors:** Pauliina Saurus, Tuomas A. Tolvanen, Sonja Lindfors, Sara Kuusela, Harry Holthöfer, Eero Lehtonen, Sanna Lehtonen

**Affiliations:** 1 Department of Pathology, University of Helsinki, Helsinki, Finland; 2 Department of Bacteriology and Immunology, University of Helsinki, Helsinki, Finland; 3 Laboratory Animal Centre, University of Helsinki, Helsinki, Finland

## Abstract

Lack of CD2-associated protein (CD2AP) in mice increases podocyte apoptosis and leads to glomerulosclerosis and renal failure. We showed previously that SHIP2, a negative regulator of the PI3K/AKT signalling pathway, interacts with CD2AP. Here, we found that the expression level and activity of SHIP2 and production of reactive oxygen species (ROS) are increased in cultured CD2AP knockout (CD2AP−/−) mouse podocytes. Oxidative stress was also increased in CD2AP−/− mouse glomeruli *in vivo*. We found that puromycin aminonucleoside (PA), known to increase ROS production and apoptosis, increases SHIP2 activity and reduces CD2AP expression in cultured human podocytes. PDK1 and CDK2, central regulators of AKT, were downregulated in CD2AP−/− or PA-treated podocytes. Downregulation of PDK1 and CDK2, ROS generation and apoptosis were prevented by CD2AP overexpression in both models. Notably, inhibition of SHIP2 activity with a small molecule inhibitor AS1949490 ameliorated ROS production in CD2AP−/− podocytes, but, surprisingly, further reduced PDK1 expression and aggravated apoptosis. AKT- and ERK-mediated signalling was diminished and remained reduced after AS1949490 treatment in the absence of CD2AP. The data suggest that inhibition of the catalytic activity of SHIP2 is beneficial in reducing oxidative stress, but leads to deleterious increase in apoptosis in podocytes with reduced expression of CD2AP.

## Introduction

Podocyte (glomerular epithelial cell) injury has been shown in many forms of clinical and experimental glomerular diseases, including, for example, focal segmental glomerulosclerosis (FSGS) and diabetic nephropathy, and is a major contributor to proteinuria^1^. Various factors may give rise to podocyte injury, including transforming growth factor beta (TGF-β) or oxidative stress resulting from excessive production of reactive oxygen species (ROS)^2–4^. In podocytes *in vitro*, high glucose induces ROS production through mitochondrial pathways and NADPH oxidase, and activates proapoptotic p38 mitogen-activated protein kinase (MAPK) and caspase-3 and induces apoptosis^2^. *In vivo*, reduction of mitochondrial oxidative stress by antioxidant treatment reduces podocyte apoptosis and renal injury^3^. Thus, therapeutic interventions aiming to reduce oxidative stress could help in preventing podocyte loss and glomerulosclerosis.

CD2-associated protein (CD2AP) is essential for podocyte function, as mice lacking CD2AP develop glomerulosclerosis and die of renal failure at the age of six to seven weeks^5^. Mutations in CD2AP have also been identified in human patients with FSGS^6–8^. TGF-β expression and apoptosis of podocytes are elevated in the absence of CD2AP in mice^9^. In line with this, CD2AP is essential for TGF-β-induced early activation of the antiapoptotic phosphatidylinositol-3-kinase/serine/threonine kinase, protein kinase B (PI3K/AKT) pathway, and lack of CD2AP intensifies the activation of the proapoptotic p38 MAPK pathway by TGF-ß1^9^. Heterozygosity of CD2AP in TGF-β1 transgenic mice increases podocyte apoptosis and renal dysfunction further pinpointing an important role for CD2AP in preventing podocyte apoptosis and kidney injury^10^. Yet, the exact molecular pathways involved in podocyte injury resulting from CD2AP-deficiency are poorly characterized, and ways to prevent the harmful process are lacking.

We found previously that CD2AP forms a complex with SH2 domain-containing inositol phosphatase 2 (SHIP2), a lipid phosphatase of the inositol 5′-phosphatase family^11^. SHIP2 negatively regulates the PI3K/AKT pathway by hydrolysing phosphatidylinositol (3,4,5)-trisphosphate (PI(3,4,5)P_3_) to phosphatidylinositol (3,4)-bisphosphate (PI(3,4)P_2_)^12^, and overexpression of SHIP2 induces podocyte apoptosis^11^. Interestingly, SHIP2 is also linked to oxidative stress in apoptosis. In hepatocytes, overexpression of a dominant-negative, inactive form of SHIP2 enhances AKT activation and reduces ROS generation and apoptosis induced by palmitate^13^. Furthermore, scavenging ROS in hepatocytes overexpressing wild-type SHIP2 ameliorates apoptosis^13^. These data suggest that inhibiting the activity of SHIP2 could provide a means to ameliorate also podocyte apoptosis induced by elevated oxidative stress.

Thus far the functional interrelationship of CD2AP and SHIP2 in podocytes has remained unclear. Here, we found that CD2AP-deficient podocytes, characterized by increased expression of TGF-β1 and apoptosis^9^, show elevated level of ROS and increased expression and activity of SHIP2. We then hypothesized that inhibition of SHIP2 activity reduces oxidative stress and apoptosis in the absence of CD2AP. Notably, we observed that inhibition of SHIP2 activity with a specific small molecule inhibitor AS1949490 reduces ROS, but contrary to our expectations, increases podocyte apoptosis in the absence of CD2AP. This suggests that inhibition of the catalytic activity of SHIP2 provides a means to reduce oxidative stress in podocytes, but aggravates rather than ameliorates podocyte injury by increasing apoptosis when CD2AP is depleted.

## Results

### Absence of CD2AP in mouse podocytes induces ROS production and apoptosis

**Figure 1:**
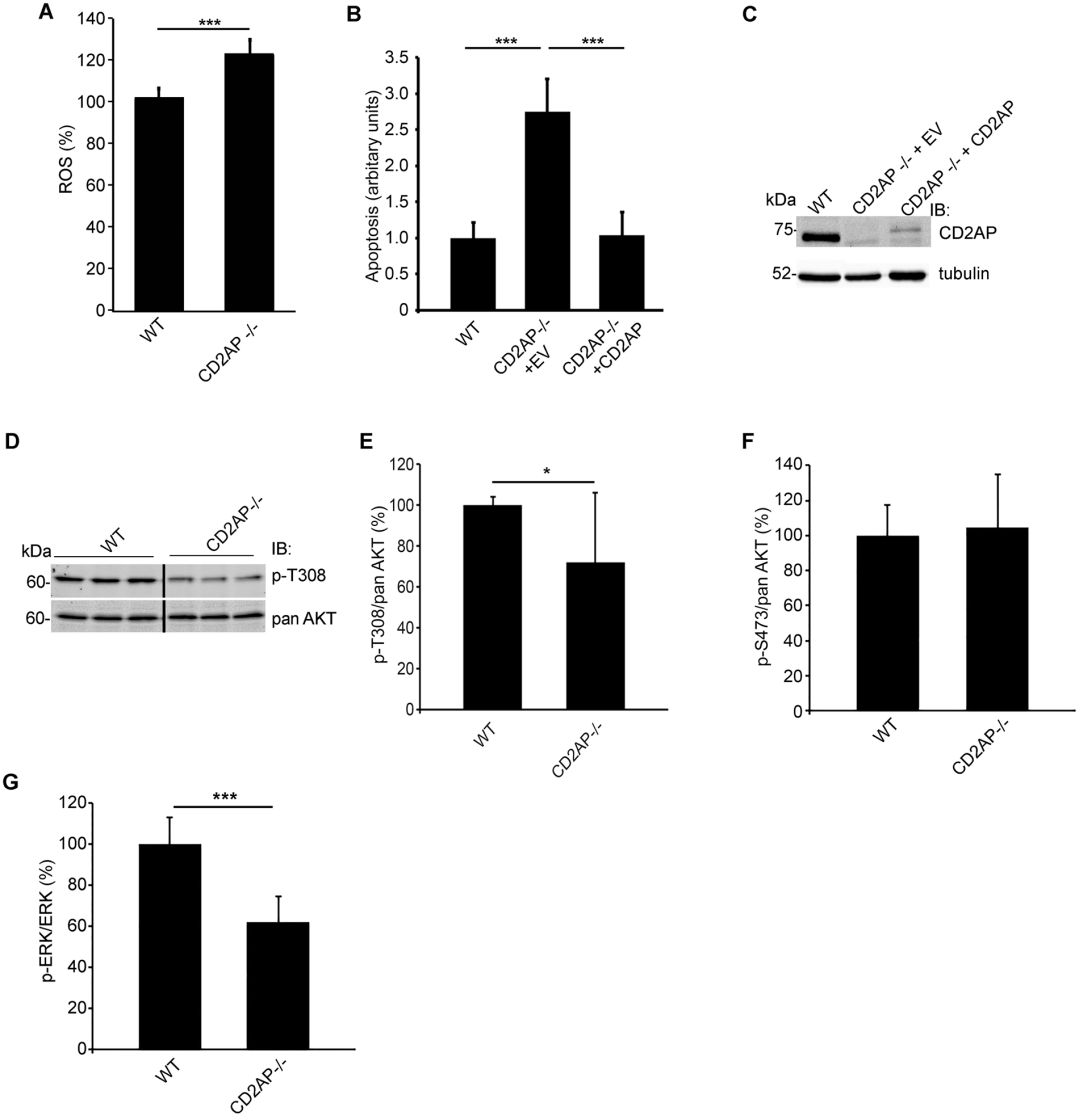
ROS generation and apoptosis are increased in CD2AP−/− podocytes. (**A**) ROS production is increased in the absence of CD2AP (CD2AP−/−) compared to wild type (WT) podocytes as observed by DCFH-DA fluorescent probe assay. Hoechst 33342 was used for normalization. (**B**) Flow cytometry with annexin V and 7-AAD double labelling indicates that absence of CD2AP induces apoptosis. Reintroduction of CD2AP into CD2AP−/− podocytes rescues CD2AP−/− podocytes from apoptosis. (**C**) Representative immunoblot of CD2AP expression in WT podocytes, and in CD2AP−/− podocytes infected with lentiviruses containing an empty vector (EV) or human CD2AP cDNA. (**D,E**) Immunoblotting and quantification reveals that phosphorylation of AKT on T308 (p-T308) is reduced in CD2AP−/− podocytes compared to WT podocytes. Total AKT (panAKT) is used for normalization. Full width blot of (D) is shown in Supplemental Fig. S1A. **(F)** In-cell Western and quantification shows no difference in the phosphorylation of AKT on S473 (p-S473) in relation to total AKT (panAKT) between WT and CD2AP−/− podocytes. Both p-S473 and panAKT were normalized to nuclear marker DRAQ5^TM^. **(G)** In-cell Western and quantification reveals that the ratio of phosphorylated ERK (p-ERK) to total ERK is significantly lower in CD2AP−/− podocytes compared to WT podocytes. Both p-ERK and total ERK were normalized to nuclear marker DRAQ5^TM^. The experiments were performed three times with three replicates in each experiment. The bars show the mean expression in arbitrary units (error bars STDEV). *p < 0.05, ***p < 0.001, Student’s t-test.

To analyse whether CD2AP and SHIP2 play a role in oxidative stress-induced apoptosis in podocytes, we first analysed whether lack of CD2AP enhances ROS production. 2′, 7′-Dichlorofluorescein diacetate (DCFH-DA) fluorescent probe assay revealed that lack of CD2AP increases ROS production by 25% (Fig. 1A), and increases apoptosis by 2.8-fold compared to wild type cells (Fig. 1B) as shown by fluorescence-activated cell sorting (FACS) with annexin V and 7-AAD, labelling apoptotic and necrotic cells, respectively. Reintroducing CD2AP back to the knockout cells, even at low level, reduced apoptosis to the same level as in wild type cells (Fig. 1B,C). In line with an increase in apoptosis, we observed that threonine 308 (T308) phosphorylation of AKT was 30% lower at basal state in the absence of CD2AP compared to wild type podocytes (Fig. 1D,E. Supplemental Fig. S1A). In contrast, we did not observe any difference in the phosphorylation of serine 473 (S473) of AKT between WT and CD2AP−/− podocytes (Fig. 1F). We also observed that phosphorylation of extracellular-signal related kinase (ERK), a member of the mitogen-activated protein kinase (MAPK) family that regulates cell survival and apoptosis (reviewed in refs 14 and 15), was significantly lower in podocytes lacking CD2AP (Fig. 1G).

### Inhibition of SHIP2 activity reduces ROS production in CD2AP−/− podocytes

**Figure 2:**
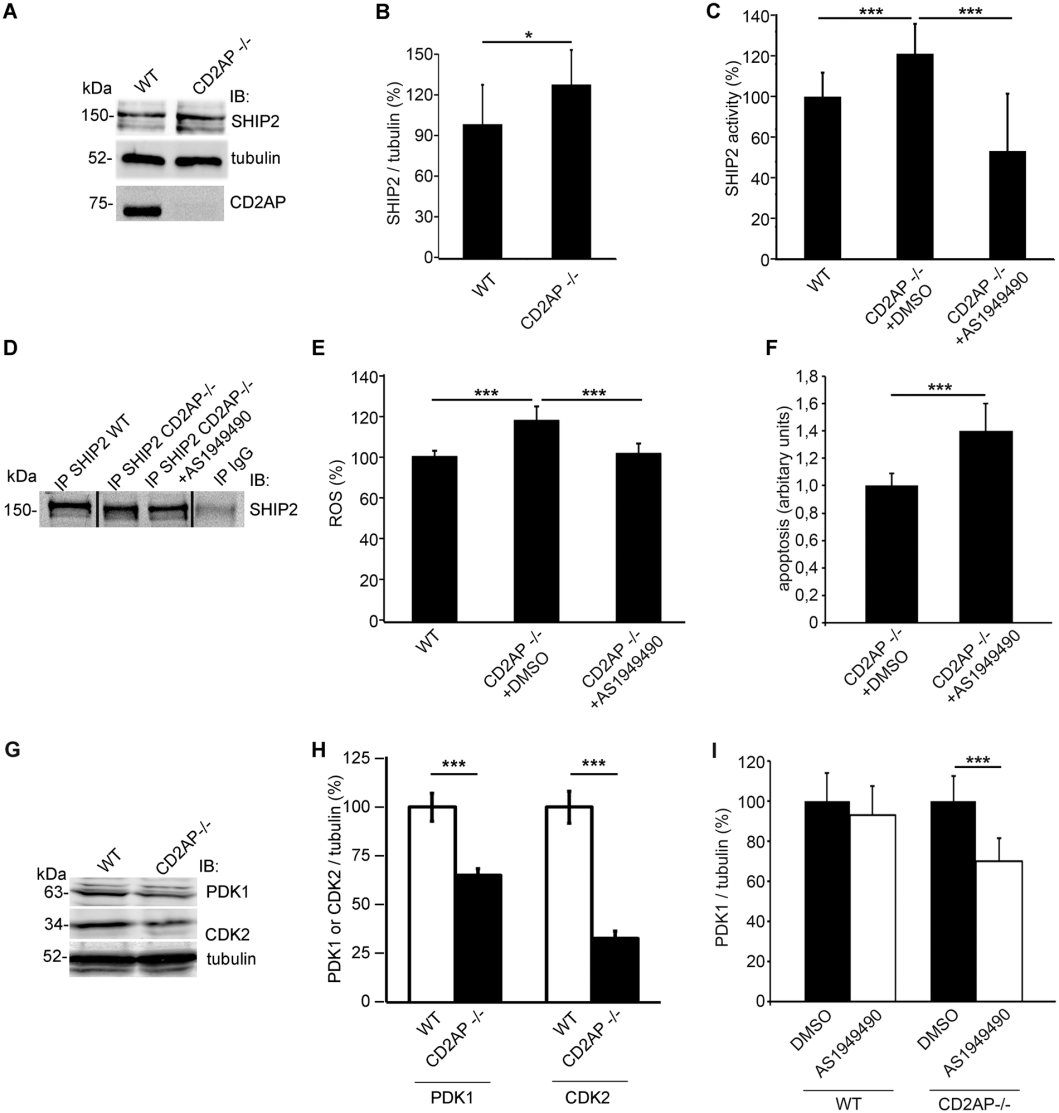
Inhibition of SHIP2 activity reduces ROS production in CD2AP knockout podocytes but does not protect from apoptosis. (**A**) Representative immunoblot for SHIP2 in wild type (WT) and CD2AP−/− podocytes. Tubulin is included as a loading control. (**B**) Quantification of SHIP2 expression level in WT and CD2AP−/− podocytes in three replicate blots as in (**A**) shows an increase in SHIP2 expression. Tubulin was used for normalisation. (**C**) SHIP2 activity is increased in the absence of CD2AP. Treatment of CD2AP−/− podocytes with SHIP2 inhibitor AS1949490 reduces SHIP2 activity. **(D)** Representative immunoblot for SHIP2 of immunoprecipitations carried out with SHIP2 IgG (IP SHIP2) or goat IgG (IP IgG) as a control from 500 µg of protein lysates prepared from WT podocytes and CD2AP−/− podocytes treated or not with AS1949490. Similar immunoprecipitations were performed to enrich SHIP2 for activity assays. Full width blot is shown in Supplemental Fig. S1B. (**E**) Inhibition of SHIP2 prevents the increase in ROS generation induced by the absence of CD2AP, as observed by DCFH-DA fluorescent probe assay. Hoechst 33342 was used for normalization. (**F**) Flow cytometry with annexin V labelling indicates that AS1949490 treatment of CD2AP−/− podocytes induces apoptosis. (**G**) Representative immunoblot for PDK1 and CDK2 in WT and CD2AP−/− podocytes. Tubulin is included as a loading control. (**H**) Quantification of PDK1 and CDK2 in three replicate blots as in (**G**) in WT and CD2AP−/− podocytes shows a decrease in PDK1 and CDK2 expression in the absence of CD2AP. (**I**) SHIP2 inhibitor AS1949490 treatment reduces the expression of PDK1 in CD2AP−/− podocytes but not in WT podocytes. The experiments were performed three times with four (**A**–**D**,**F**–**I**) or 32 (**E**) replicates in each experiment. The bars show the mean expression in arbitrary units (error bars STDEV). *p < 0.05, ***p < 0.001, Student’s t-test.

We next defined whether CD2AP and SHIP2 are functionally interrelated, and found that the expression of SHIP2 was increased by 30% in CD2AP−/− podocytes (Fig. 2A,B). In line with this, SHIP2 activity was 26% higher in CD2AP−/− podocytes compared to wild type cells (Fig. 2C). Treatment of CD2AP−/− podocytes with AS1949490, a SHIP2-specific small molecule inhibitor^16^, reduced SHIP2 activity by 33% compared to wild type podocytes, and by 47% compared to CD2AP−/− podocytes without treatment (Fig. 2C). The differences detected in the activities were not due to different amounts of immunoprecipitated SHIP2 used for the malachite green phosphate assay (Figs 2D and S1B). AS1949490 also reduced ROS production in CD2AP−/− podocytes from 122% back to the level observed in wild type cells (Fig. 2E). Contrary to our expectations, inhibition of SHIP2 did not ameliorate apoptosis caused by lack of CD2AP but rather increased it by 40% (Fig. 2F).

To define the reason for this, we investigated the expression of 3-phosphoinositide-dependent protein kinase 1 (PDK1) and cyclin-dependent kinase 2 (CDK2), both shown to induce apoptosis in podocytes when knocked down^17, 18^. Interestingly, the expression of PDK1 was downregulated by 35% and CDK2 by 65% in CD2AP−/− podocytes when compared to wild type cells (Fig. 2G,H). Notably, AS1949490 treatment led to further downregulation of PDK1 expression in CD2AP−/− podocytes but not in wild type podocytes (Fig. 2I). Collectively, PDK1 and CDK2 are downregulated in the absence of CD2AP and may contribute to increased apoptosis. Inhibition of SHIP2 further reduces the total amount of PDK1 in the absence of CD2AP, which may aggravate apoptosis.

**Figure 3:**
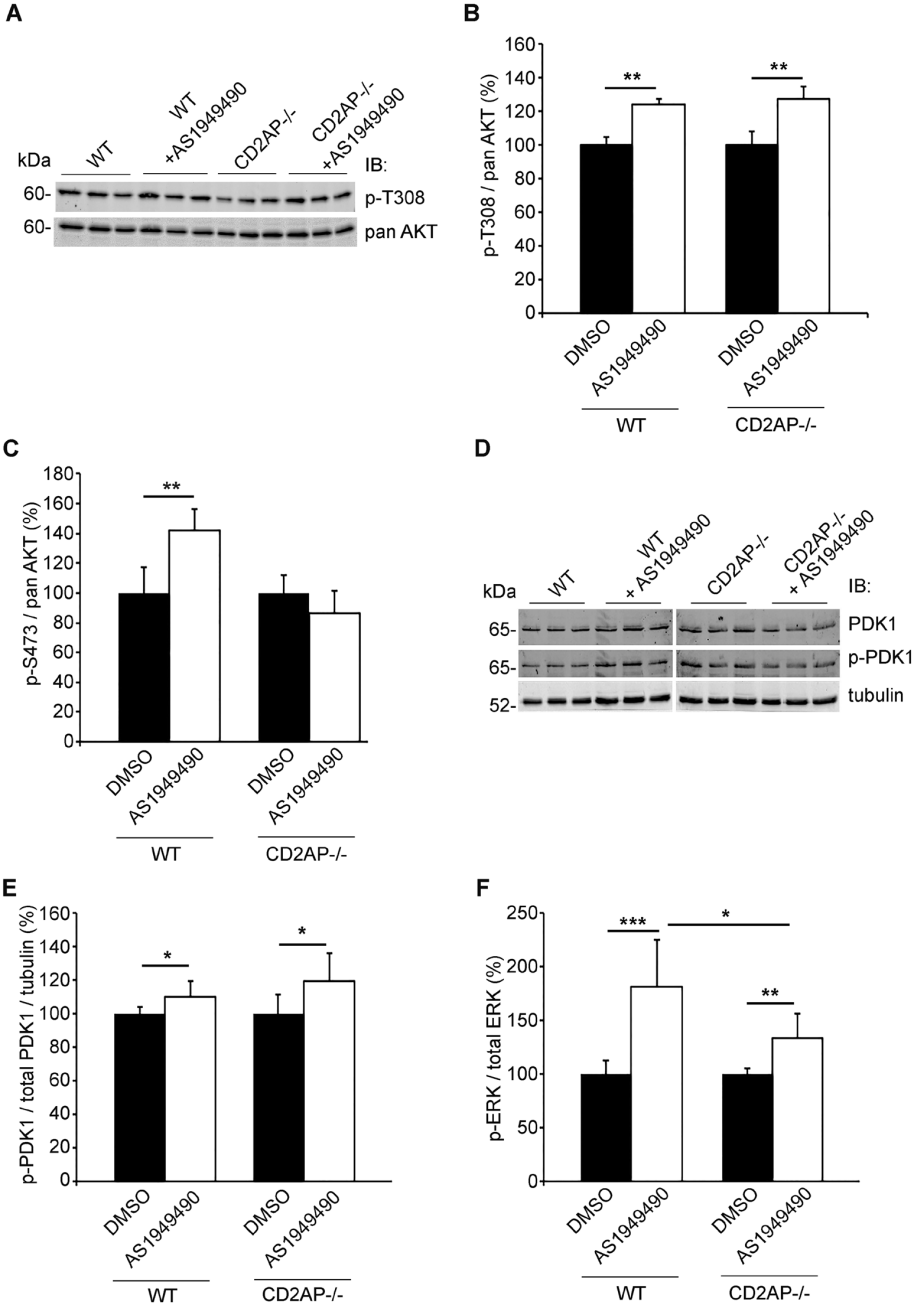
SHIP2 inhibition diminishes activation of AKT and ERK signalling pathways in CD2AP-deficient podocytes. (**A**) Representative immunoblot used for quantifying T308 phosphorylation of AKT in WT and CD2AP−/− podocytes treated or not with AS1949490. (**B**) Quantification of three replicate immunoblots as in (**A**) reveals that AS1949490 treatment increases T308 phosphorylation of AKT (p-T308) in both WT and CD2AP−/− podocytes. **(C)** In-cell Western and quantification shows that AS1949490 treatment increases S473 phosphorylation of AKT (p-S473) in WT podocytes but not in CD2AP−/− podocytes. Phosphorylated AKT (S473) and total AKT (panAKT) were both normalized to nuclear marker DRAQ5^TM^. **(D)** Representative immunoblot used for quantifying p-PDK1 in WT and CD2AP−/− podocytes treated or not with AS1949490. **(E)** Quantification of three replicate immunoblots as in (D) reveals that AS1949490 treatment increases relative phosphorylation of PDK1 in both WT and CD2AP−/− podocytes. p-PDK1 and total PDK1 were normalized to tubulin before calculation of the ratio. **(F)** In-cell Western and quantification reveals that AS1949490 treatment increases the phosphorylation of ERK (p-ERK) in both WT and CD2AP−/− podocytes, but the response remains significantly lower in the absence of CD2AP. The experiments were performed three times with three replicates in each experiment. The bars show the mean expression in arbitrary units (error bars STDEV). *p < 0.05, **p < 0.01, ***p < 0.001, Student’s t-test.

To further investigate the signalling pathways associated with an increase in apoptosis, we determined the activity of AKT and ERK after AS1949490 treatment. T308 phosphorylation of AKT, shown to be mediated by PDK1^19^, was increased in both WT and CD2AP−/− podocytes after the AS1949490 treatment (Fig. 3A,B). In contrast, AS1949490 increased S473 phosphorylation of AKT only in WT podocytes but not in CD2AP−/− podocytes (Fig. 3C). As inhibition of SHIP2 increases the amount of PI(3,4,5)P_3_ which activates PDK1^19–21^, we investigated whether inhibition of SHIP2 increases the activity of PDK1. AS1949490 treatment increased the phosphorylation of serine 241 residue of PDK1, mandatory for the activation of PDK1^22^, in both WT and CD2AP−/− podocytes (Fig. 3D,E). Phosphorylation of ERK was increased in both cell types after AS1949490 treatment but significantly less in CD2AP−/− podocytes compared to WT podocytes (Fig. 3F).

### Lack of CD2AP in mice *in vivo* enhances ROS production in glomeruli

**Figure 4:**
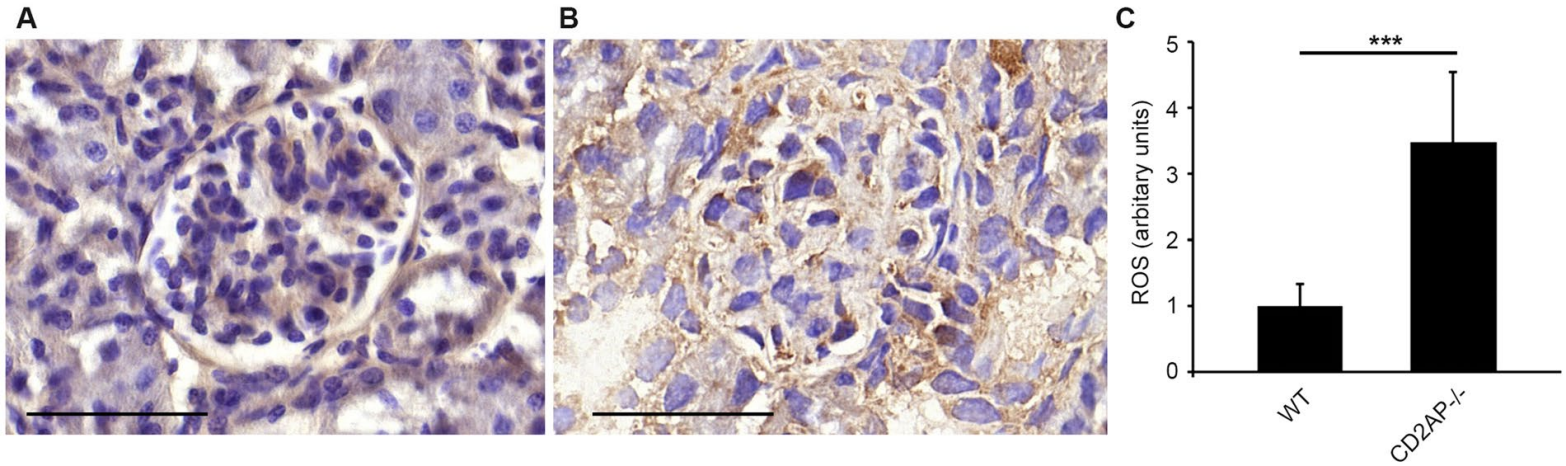
Absence of CD2AP in mouse kidneys leads to an increase in ROS. (**A**) Representative image of a 3 weeks old wild type mouse kidney section stained with anti-8-OHdG IgG. (**B**) Representative image of a 3 weeks old CD2AP−/− mouse kidney section stained with anti-8-OHdG IgG. (**C**) Quantification reveals 3.5-fold increase in 8-OHdG staining in glomeruli of CD2AP−/− mice reflecting oxidative damage to DNA and RNA. Scale bar (**A**,**B**): 40 µm. In (**C**), the bars show the difference in arbitrary units between relative mask areas quantified with the HistoQuant module (error bars SEM). ***p < 0.001, Student’s t-test.

As lack of CD2AP increased oxidative stress in cultured podocytes (Fig. 1A), we stained kidneys of three weeks old wild type and CD2AP−/− mice for 8-hydroxyguanosine (8-OHdG), a marker of oxidative damage (Fig. 4A,B). Quantification of the signal in glomeruli revealed 3.5-fold increase in ROS generation in the absence of CD2AP (Fig. 4C).

### PA-treatment downregulates CD2AP, upregulates SHIP2 and increases ROS production and apoptosis in cultured human podocytes

**Figure 5:**
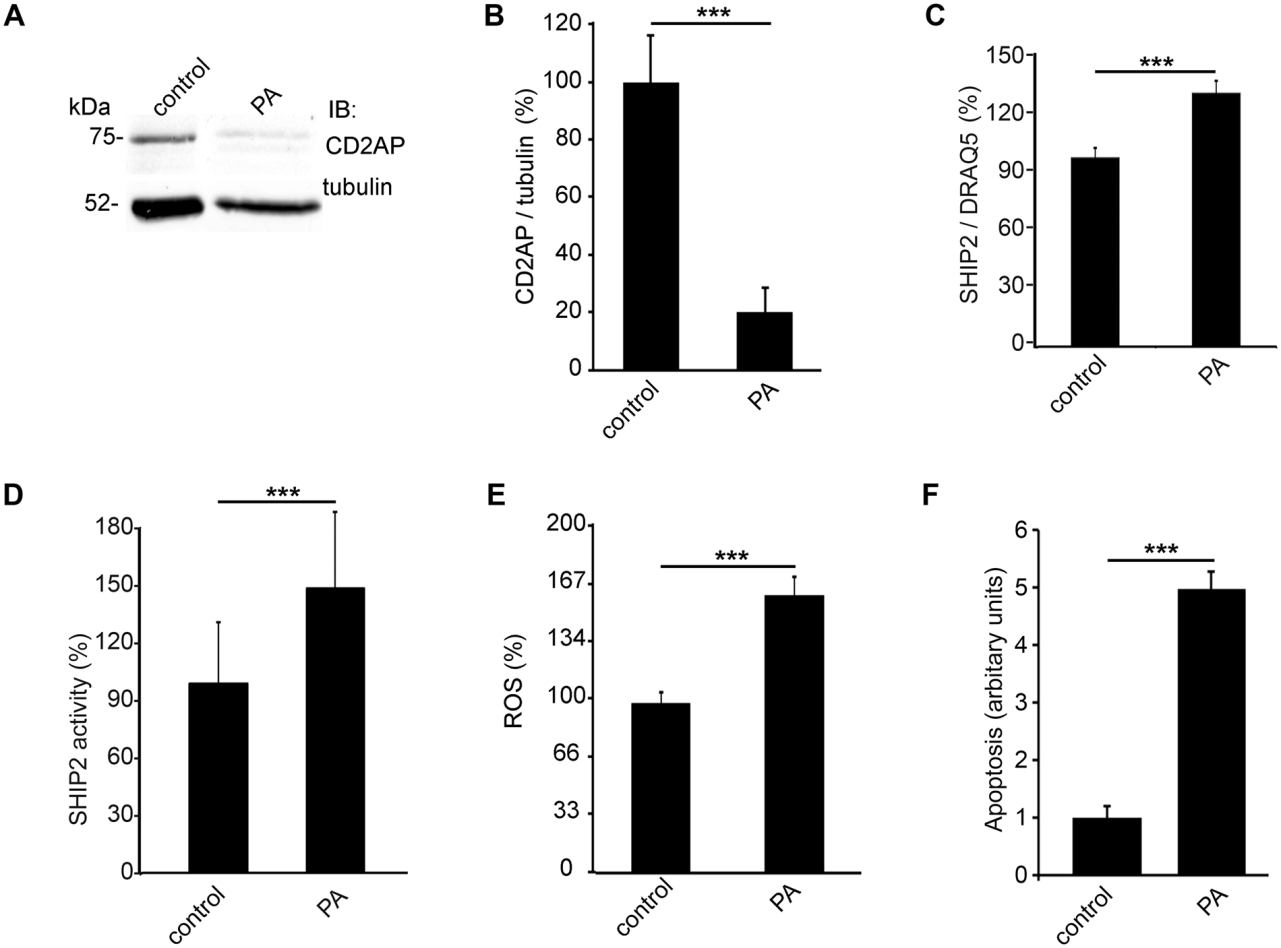
PA-treatment decreases CD2AP expression and increases SHIP2 activity, ROS production and apoptosis in cultured human podocytes. (**A**) Representative immunoblot for CD2AP after PA-treatment. Tubulin is included as a loading control. (**B**) Quantification of CD2AP after PA-treatment in immunoblots like in (**A**) shows a decrease in CD2AP expression. (**C**) Quantification of SHIP2 expression in podocytes treated with PA by In-Cell Western shows that the expression of SHIP2 is increased by PA-treatment. DRAQ5^TM^ was used for normalization. (**D**) PA-treatment increases SHIP2 activity in human podocytes. (**E**) PA increases ROS generation as visualized by an increase in the intensity of DCFH-DA fluorescent probe. Hoechst 33342 was used for normalization. (**F**) Flow cytometry of cultured human podocytes stained with annexin V and 7-AAD double labelling shows that PA-treatment increases podocyte apoptosis. The experiments were performed three times with four (**B**,**D**,**F**), 24 (**C**) or 48 (**E**) replicates in each experiment. The bars (**B**–**F**) show the mean expression in arbitrary units (error bars STDEV). ***p < 0.001, Student’s t-test.

Since puromycin aminonucleoside (PA), a highly potent podocyte toxin, is known to cause podocyte injury by induction of DNA damage mediated via ROS^23^ and to increase apoptosis in a time- and dose-dependent manner^24^, we treated differentiated human podocytes in culture with PA and analysed the effect on CD2AP and SHIP2. PA-treatment of podocytes for 48 hours decreased the expression level of CD2AP by 80% (Fig. 5A,B), and increased SHIP2 expression by 34% (Fig. 5C) and its phosphatase activity by 45% (Fig. 5D). PA increased ROS generation by 60% as shown by DCFH-DA fluorescent probe assay (Fig. 5E). FACS analysis with annexin V and 7-AAD double labelling indicated that the level of podocyte apoptosis was increased by 5-fold after PA-treatment (Fig. 5F) confirming previous findings^24^.

### SHIP2 inhibition in PA-treated cultured human podocytes does not reduce ROS production or apoptosis

**Figure 6:**
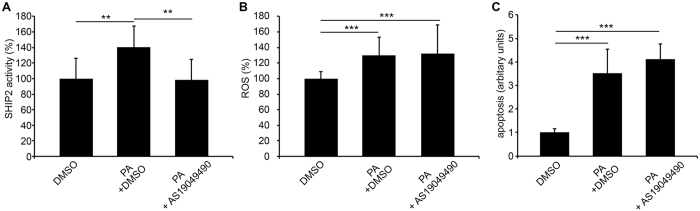
Inhibition of SHIP2 activity does not reduce PA-induced ROS generation or protect cultured human podocytes from apoptosis. (**A**) PA increases SHIP2 activity of cultured human podocytes. Simultaneous treatment of podocytes with PA and SHIP2 inhibitor AS1949490 prevents PA-induced increase in SHIP2 activity. (**B**) PA induces ROS generation in cultured human podocytes as observed by DCFH-DA fluorescent probe assay. Hoechst 33342 was used for normalization. Treatment of human podocytes with SHIP2 inhibitor AS1949490 does not prevent PA-induced increase in ROS generation. (**C**) PA induces apoptosis in cultured human podocytes as visualized by annexin V staining and FACS analysis. Treatment of human podocytes with SHIP2 inhibitor AS1949490 does not prevent PA-induced increase in apoptosis. The experiments were performed three times with four (**A**), 32 (**B**) or three (**C**) replicates in each experiment. The bars show the mean expression in arbitrary units (error bars STDEV). **p < 0.01, ***p < 0.001, Student’s t-test.

We further tested whether inhibition of SHIP2 reduces ROS production and apoptosis induced by PA. We first confirmed that AS1949490 treatment prevents the increase in the activity of SHIP2 caused by PA (Fig. 6A). As expected, the activity of SHIP2 remained at the level of the control when podocytes were treated simultaneously with PA and AS1949490 for 48 hours (Fig. 6A). However, this did not prevent an increase in ROS production (Fig. 6B) or rescue podocytes from apoptosis (Fig. 6C).

### CD2AP overexpression reduces ROS production and apoptosis induced by PA in cultured human podocytes

**Figure 7:**
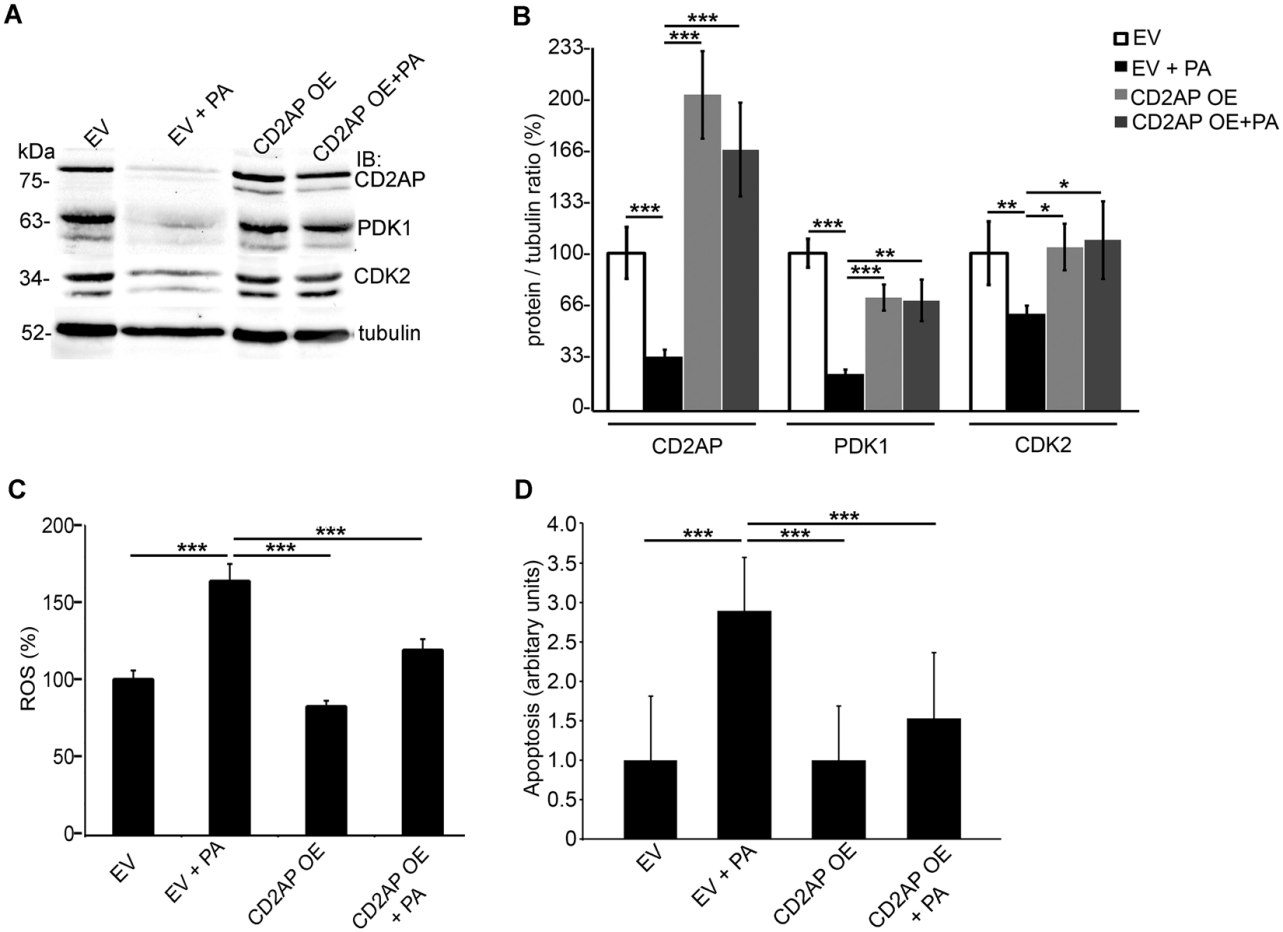
CD2AP overexpression restores PDK1 and CDK2 expression and reduces ROS generation and podocyte apoptosis induced by PA in cultured human podocytes. (**A**) Representative immunoblot for CD2AP, PDK1 and CDK2 in cultured human podocytes infected with lentiviruses containing an empty vector (EV) or human CD2AP cDNA (CD2AP OE), and with or without PA-treatment. Tubulin is included as a loading control. (**B**) Quantification of CD2AP, PDK1, and CDK2 expression level in Western blots as in (**A**) shows that PA-treatment reduces the expression of CD2AP, PDK1, and CDK2 and that CD2AP overexpression prevents PA-induced downregulation of CD2AP. Overexpression of CD2AP alone does not affect the expression of PDK1 or CDK2. (**C**) Overexpression of CD2AP prevents PA-induced increase in ROS generation as visualized by DCFH-DA fluorescent probe assay. Overexpression of CD2AP alone does not affect ROS generation. Hoechst 33342 was used for normalization. (**D**) Flow cytometry of cultured human podocytes stained with annexin V and 7-AAD confirms that overexpression of CD2AP protects podocytes from PA-induced apoptosis. The experiments were performed three times with three (**A**–**B**,**D**) or 24 (**C**) replicates in each experiment. The bars (b–d) show the mean expression in arbitrary units (error bars STDEV). *p < 0.05, **p < 0.01, ***p < 0.001, Student’s t-test.

To define whether CD2AP is able to rescue podocytes from the effects of PA, including downregulation of PDK1 and CDK2^17^, we overexpressed CD2AP simultaneously with PA-treatment of cultured human podocytes. PA-treatment reduced the expression of PDK1 by 80% and CDK2 by 40% compared to the empty vector (Fig. 7A,B), and overexpression of CD2AP significantly prevented PA-induced downregulation of both PDK1 and CDK2 (Fig. 7A,B). Overexpression of CD2AP alone had no significant effect on the expression level of any of the analysed proteins (Fig. 7A,B).

We then defined whether reintroduction of CD2AP rescues podocytes from PA-induced ROS production and apoptosis. PA-treatment increased ROS production by 64% compared to empty vector-transfected podocytes (Fig. 7C), and indeed, overexpression of CD2AP simultaneously with PA-treatment significantly reduced the increase in ROS generation, leading to only 19% increase (Fig. 7C). PA-treatment increased apoptosis by 2.9-fold compared to non-treated podocytes transfected with empty vector (Fig. 7D). The apoptosis rate of PA-treated podocytes overexpressing CD2AP was increased only by 1.5-fold, which did not significantly differ from the rate of apoptosis observed in empty vector-transfected cells (Fig. 7D). Overexpression of CD2AP alone caused no significant difference in ROS generation or apoptosis compared to empty vector-transfected podocytes (Fig. 7C,D). Collectively, the data show that reintroduction of CD2AP back to podocytes treated with PA, showing downregulation of CD2AP, is able to revert an increase in ROS production and apoptosis caused by PA.

### PA-treatment reduces CD2AP and increases SHIP2 expression in PA-induced nephrotic rats

**Figure 8:**
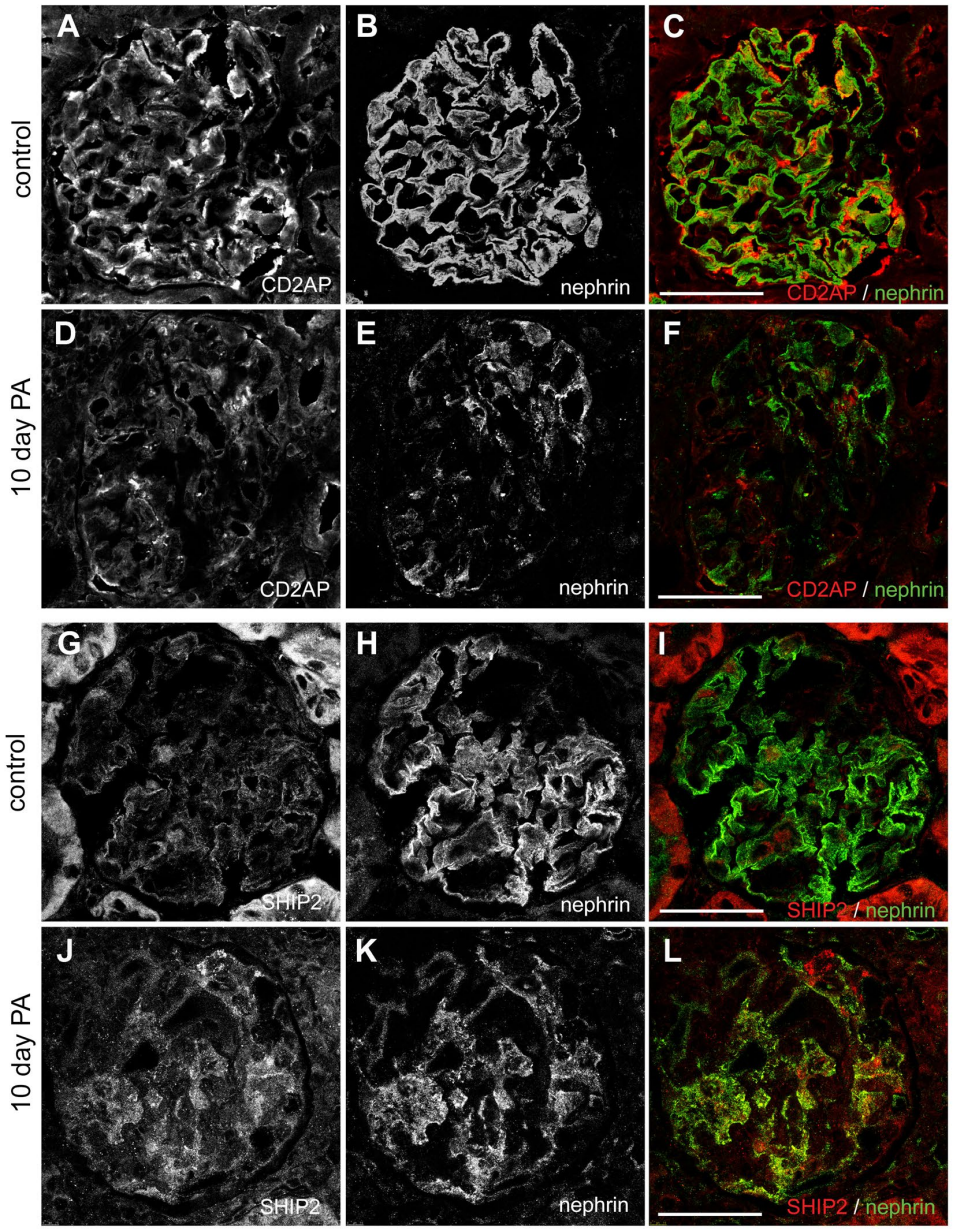
CD2AP expression appears to be reduced and SHIP2 expression increased in kidneys of PA-induced nephrotic rats. (**A**–**C**) In normal rat glomerulus CD2AP is expressed in podocytes and partially co-localizes with nephrin. (**D**–**F**) In PA-induced nephrotic rats 10 days after PA administration, both CD2AP and nephrin appear weak and show patch-like accumulation of the signal. (**G**–**I**) In normal rat glomerulus weak signal for SHIP2 is observed in podocytes as visualized by double labelling for nephrin. (**J**–**L**) In PA-induced nephrotic rats 10 days after PA administration, SHIP2 staining appears in diffuse, patch-like accumulations similarly as nephrin. Scale bar: 20 µm.

To define whether PA-treatment leads to downregulation of CD2AP and upregulation of SHIP2 *in vivo*, we immunostained kidneys of rats with PA-induced nephrosis. The rats had significantly increased proteinuria 9 days after PA administration^25^, and as previously shown^25^, greatly diminished expression of nephrin 10 days after induction of nephrosis (Fig. 8B,E,H,K). Double immunofluorescence staining for nephrin and CD2AP or SHIP2 revealed that the expression of CD2AP appears diminished (Fig. 8A–F) and SHIP2 increased (Fig. 8G–L) in podocytes in rats with PA-induced nephrosis, confirming the data obtained in cultured cells.

## Discussion

CD2AP-deficient podocytes are more prone to undergo apoptosis than wild type podocytes^9^. Here, we show that lack of CD2AP in podocytes induces oxidative stress, which may be one of the contributing factors leading to an increase in apoptosis. We also observed that the activity of SHIP2, an interaction partner of CD2AP^11^, is increased in the absence of CD2AP. Notably, we further found that inhibition of SHIP2 activity ameliorates oxidative stress caused by lack of CD2AP. However, SHIP2 inhibition alone was not able to rescue CD2AP-deficient podocytes from apoptosis.

We showed previously that CD2AP associates with SHIP2^11^, and here we found that SHIP2 expression and activity are upregulated in CD2AP−/− podocytes. Together these data suggest that CD2AP has an inhibitory role on SHIP2. Our previous data revealed that overexpression of SHIP2 in podocytes reduces AKT activation and enhances apoptosis^11^, suggesting that increased expression of SHIP2 could contribute to increased apoptosis in CD2AP−/− podocytes. Elevated SHIP2 increases apoptosis also in neuronal cells in SHIP2 overexpressing mice^26^ and hepatocytes^13^. In line with the elevated activity of SHIP2 in CD2AP−/− podocytes, we observed that the activity of the AKT and ERK cell survival pathways at basal state were reduced (as visualized by reduced AKT and ERK phosphorylation) in the absence of CD2AP reflecting the increased rate of apoptosis. This is in line with earlier literature showing that CD2AP interacts with p85 subunit of PI3K and, together with podocyte proteins nephrin and podocin, stimulates the PI3K/AKT pathway^27^. The data are also consistent with a previous report revealing reduced base-line activation of ERK in the absence of CD2AP^28^. Reduced ERK activation may be due to an increase in SHIP2 expression, as a previous study showed decreased insulin-stimulated ERK activation in SHIP2 overexpressing cells^12^.

**Figure 9:**
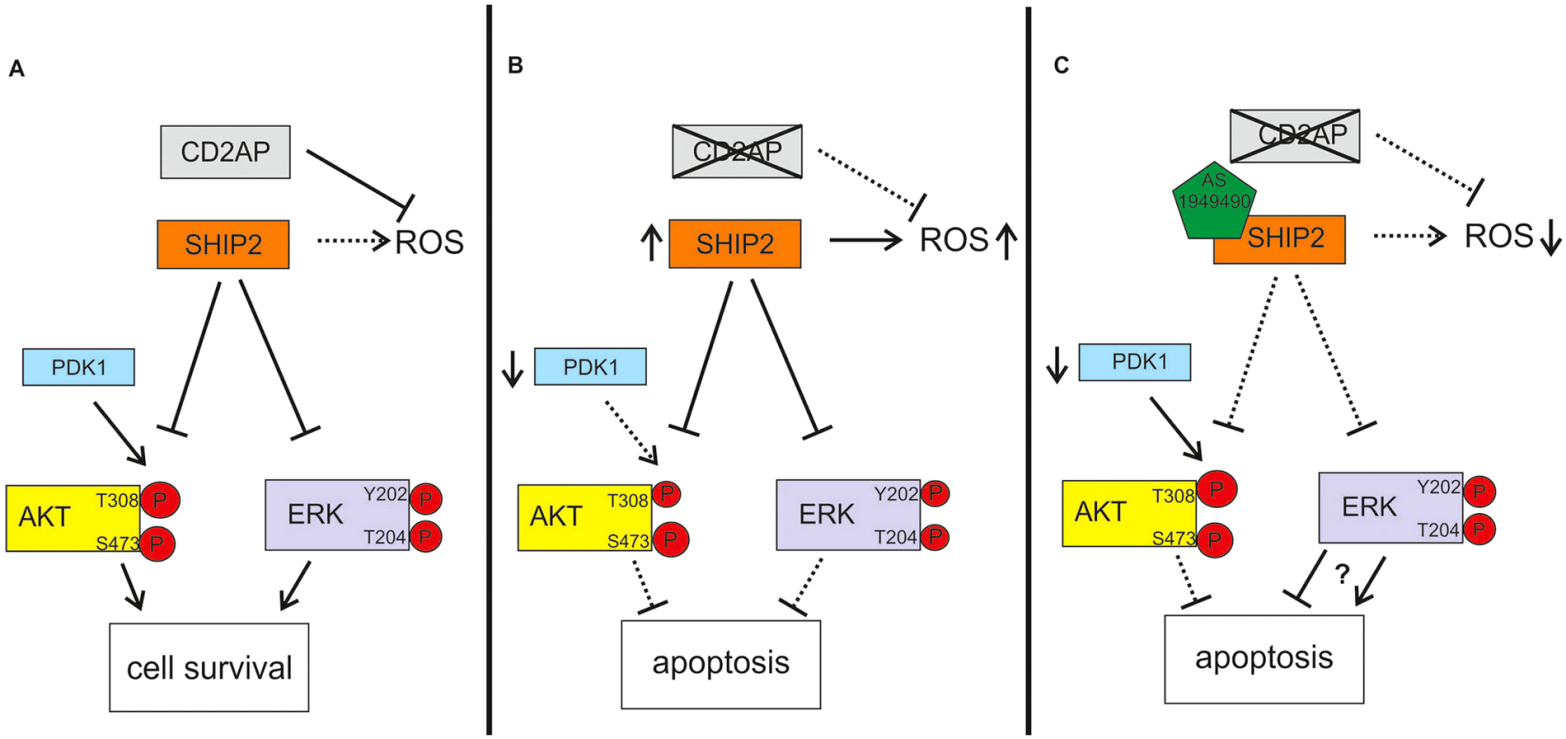
Schematic model illustrating the effect of SHIP2 inhibition in CD2AP-deficient podocytes. (**A**) Wild type podocytes in basal state. CD2AP associates with SHIP2. CD2AP inhibits the production of ROS, and SHIP2 negatively regulates the pathways leading to AKT and ERK phosphorylation. The balance between the prosurvival and proapoptotic signals is maintained. **(B)** CD2AP−/− podocytes in basal state. The expression and activity of SHIP2 and generation of ROS are increased in the absence of CD2AP. Increased SHIP2 activity leads to low phosphorylation level of AKT and ERK. The expression of PDK1 is downregulated contributing to degreased phosphorylation of T308 of AKT. Decrease in prosurvival signalling manifests as increased apoptosis of the cells. **(C)** Inhibition of SHIP2 activity with AS1949490 in CD2AP−/− podocytes. Inhibition of the activity of SHIP2 leads to reduced generation of ROS. AS1949490 attenuates the potential of SHIP2 to negatively regulate AKT and ERK activity, yet the phosphorylation of S473 of AKT does not increase. Despite of low expression level of PDK1, the phosphorylation of T308 of AKT increases. As phosphorylation of both sites of AKT is required for its full activity, remains the balance between prosurvival and proapoptotic signalling disrupted and podocytes undergo apoptosis. It is also possible that increased ERK activation may contribute to an increase in apoptosis (see Discussion for details).

Interestingly, we found that lack of CD2AP reduces the expression of PDK1, a central activator of AKT^19^, providing a further link between CD2AP and cell survival signalling (Fig. 9). Pancreatic β cells depleted of PDK1 are more prone to undergo apoptosis^29^ and in line with this, knockdown of PDK1 in podocytes increases apoptosis by inhibiting antiapoptotic and stimulating proapoptotic pathways^18^. Here, we observed that lack of CD2AP also reduces the expression of CDK2, a cell cycle regulatory protein previously shown to activate AKT during cell cycle progression^30^. Notably, knockdown of CDK2 downregulates PDK1, decreases activation of AKT and increases apoptosis^17^. Furthermore, knockdown of PDK1 downregulates CDK2 and thus proposes a regulatory loop between the proteins^17^. The data suggest that PDK1 and CDK2 play central roles in CD2AP-mediated control of the AKT cell survival pathway, but the exact mechanisms leading to their downregulation in the absence of CD2AP require further studies.

Excess generation of ROS has been shown to lead to apoptosis and progression of kidney injury^31, 32^. Our study shows that an increase in ROS may be one of the mediators of podocyte injury in the absence of CD2AP, as we observed increased ROS in CD2AP−/− kidneys at the age of three weeks, which coincides with an increase in podocyte apoptosis and the development of albuminuria and glomerular lesions reminiscent of FSGS^9^. A recent study proposed that podocytes bind plasminogen and activate it, which then evokes production of superoxide anion (a type of ROS) that further contributes to podocyte injury^33^. Interestingly, the authors found increased concentrations of plasminogen in the urine of CD2AP knockout mice which could lead to increased ROS in CD2AP-deficient kidneys^33^, in line with our findings. Resembling CD2AP-deficient podocytes, PA-treatment leads to downregulation of CD2AP and PDK1 (this study) and increases ROS generation^23^.

The key finding of this study is that inhibition of SHIP2 activity with a small-molecule inhibitor AS1949490 in the absence of CD2AP reduces ROS generation (Fig. 9). AS1949490 is highly specific towards SHIP2 and does not inhibit other phosphatases that regulate the PI3K-pathway such as SHIP1, a close homologue of SHIP2, or phosphatase and tensin homolog (PTEN), a lipid 3′-phosphatase^16^. Previous studies in hepatocytes indicated that catalytically inactive form of SHIP2 protects against palmitate or high glucose -induced excessive production of ROS and apoptosis^13, 34^. In the absence of CD2AP, however, inhibition of SHIP2 activity alone was not able to rescue podocytes from apoptosis, but rather increased it. This could be due to the fact that SHIP2 inhibition increased only T308 but not S473 phosphorylation of AKT in CD2AP-deficient cells (Fig. 9), which is not enough to fully activate AKT^35^. Phosphorylation of T308 may sound contradictory as phosphorylation of this site occurs by PDK1^19^ and PDK1 is downregulated in the absence of CD2AP. However, as the catalytic activity of SHIP2 is to hydrolyse PI(3,4,5)P_3_ to PI(3,4)P_2_, SHIP2 inhibition is expected to lead to an increase in PI(3,4,5)P_3_ in cells similarly as after SHIP2 knockdown^36^. Increase in PI(3,4,5)P_3_, in turn, may lead to activation of PDK1 (as we observed here), in line with studies showing that PDK1 is activated by both PI(3,4,5)P_3_ and PI(3,4)P_2_^19–21^, but that it binds PI(3,4,5)P_3_ more strongly than PI(3,4)P_2_^21^. PI(3,4,5)P_3_ also binds AKT, apparently altering its conformation, and thereby enhances its binding to PDK1 contributing to an increase in T308 phosphorylation^37^. Furthermore, even though AS1949490 treatment slightly increased the phosphorylation of ERK in the absence of CD2AP, its activity remained lower compared to WT podocytes treated with AS1949490. In a similar fashion, a previous study showed an increase in AKT and ERK phosphorylation by AS1949490 in lymphatic endothelial cells^38^. Interestingly, the authors found that knockdown of SHIP2 in these cells, contrary to expectations, increased apoptosis^38^, resembling our finding here that SHIP2 inhibition in the absence of CD2AP increases apoptosis. As ERK activation may also trigger apoptosis^14^ and ERK activation has been associated with podocyte injury^39^, it is also possible that SHIP2 inhibition in the absence of CD2AP triggers proapoptotic pathways leading to reduced cell survival (Fig. 9).

The inability of SHIP2 inhibition to rescue CD2AP-deficient podocytes from apoptosis does not rule out the potential of SHIP2 inhibitors to reduce ROS and apoptosis in proteinuric diseases presenting normal expression levels of CD2AP. Increased levels of ROS have been observed, for example, in podocytes in diabetic kidney disease^2^. Our previous finding that SHIP2 is upregulated in glomeruli in diabetic rodent models^11^ together with this study suggests that elevated SHIP2 could contribute to an increase in ROS in podocytes in diabetes. In future studies it will be of interest to define whether inhibition of SHIP2 in diabetic kidney disease reduces the increased levels of ROS, and thereby potentially prevents or slows down the progression of the disease.

Reduced expression of CD2AP apparently associates with multiple pathways leading to podocyte injury, shown here with both CD2AP-deficient podocytes and the PA model of podocyte injury. The latter was our model of choice to be used side by side with CD2AP−/− podocytes, as the PA-induced nephrosis in rats is a commonly used model for FSGS^40^, and the glomerular changes observed in CD2AP knockout mice resemble those observed in human FSGS^5^. In addition, mutations in CD2AP, detected in a subgroup of patients with FSGS, lead to reduced expression of CD2AP^6–8^. The molecular changes, including decrease of CD2AP, PDK1 and CDK2 expression, and increase of SHIP2 activity, oxidative stress and apoptosis, were shared between the two models. In the harsh PA injury model, apparently affecting a multitude of proteins in addition to CD2AP, inhibition of SHIP2 did not rescue podocytes from excess ROS generation and apoptosis, whereas overexpression of CD2AP did. In CD2AP−/− podocytes, SHIP2 inhibition did reduce ROS but aggravated apoptosis. Resembling this, we observed previously increased activity of β-catenin in podocytes in the absence of CD2AP, but found that inhibition of the Wnt/β-catenin pathway did not ameliorate but further aggravated kidney injury^41^. These data reinforce the central role of CD2AP as a regulator of multiple pathways in podocytes and reveal that inhibition of the harmful pathways may have unexpected adverse effects.

Collectively, SHIP2 inhibitors may be effective in preventing excessive generation of ROS. However, this may not be a suitable approach to treat kidney diseases associated with reduced expression or lack of CD2AP. Further studies are necessary to define the applicability of SHIP2 inhibition as a part of therapy in kidney diseases presenting with oxidative stress, such as diabetic kidney disease.

## Materials and Methods

### Cell culture

Mouse wild type and CD2AP−/− podocytes, HEK 293FT cells (Sigma-Aldrich, St. Louis, MO) and conditionally immortalized human podocytes (AB 8/13) were maintained as decribed^42^. Media and foetal bovine serum (FBS) were from Sigma-Aldrich, ITS from Gibco (Paisley, UK) and ultraglutamine from Lonza (Basel, Switzerland).

### Measurement of ROS production

Cells cultured in 96-well plates were treated where indicated with 10 µM AS1949490 (Tocris Biosciences, Bristol, UK), a small molecule inhibitor of SHIP2^16^, for 24 hours. Cells were washed with phenol red-free RPMI 1640 (Sigma-Aldrich) and incubated with 50 µM 2′,7′-Dichlorofluorescein diacetate (DCFH-DA) fluorescent probe and 2 µM Hoechst 33342 (Fluka, Sigma Aldrich) (100 μl/well) at 37 °C for 60 min. After washing twice with phenol red-free RPMI 1640, the fluorescence intensity was measured at 485 nm excitation and 525 nm emission wavelength with a Fluoroskan Ascent FL lumino/fluorometer (Thermo Fisher Scientific, Waltham, MA, USA).

### Induction and detection of apoptosis

Differentiated human podocytes were exposed to PA (Sigma-Aldrich, 50 µg/ml) for 48 hours. After 24 h, podocytes were treated with 10 µM AS1949490 or DMSO (diluent) as a control. CD2AP−/− and wild type podocytes were treated with 10 µM AS1949490 or DMSO for 24 h prior to detection of apoptosis. Apoptosis was detected by flow cytometry using annexin V-FITC and 7-AAD double staining with FACSAria (BD Biosciences, Franklin Lakes, NJ) or CyAn ADP (Beckman Coulter, Brea, CA). Cells positive for annexinV-FITC and negative for 7-AAD were deemed apoptotic. A total 1 × 10^4^ cells were detected by FACS in each sample.

### Immunoblotting and antibodies

Immunoblotting was performed as described previously^11^. Primary antibodies used were mouse monoclonal anti-8-hydroxyguanosine (8-OHdG) (15A3), rabbit polyclonal anti-CD2AP H-290, mouse monoclonal anti-CDK2 and goat anti-SHIP2 I-20 IgGs (Santa Cruz Biotechnology, Dallas, Texas, USA), rabbit polyclonal, anti-phospho-PDK1 (Ser241), anti-PDK1, anti-phospho-p44/p42 MAPK (T202/Y204), anti-p44/p42 MAPK (referred to as ERK), and anti–phospho-AKT (Thr308) IgGs, and mouse monoclonal anti-phospho-AKT (Ser473) IgG (Cell Signaling Technology, Danvers, MA), mouse monoclonal anti–Pan AKT IgG (R&D Systems, Minneapolis, MN) and mouse monoclonal anti-tubulin IgG (Sigma-Aldrich). Rabbit polyclonal anti-CD2AP IgG raised against mouse CD2AP was described previously^43^. Alexa Fluor 680 (Invitrogen, Carlsbad, CA, USA) and IRDye 800 (LI-COR, Lincoln, NE) donkey anti-rabbit, anti-goat or anti-mouse IgGs were used as secondary antibodies. Detection and quantification was performed with an Odyssey Infrared Imager (LI-COR). Full-length lanes of blots probed with antibodies against SHIP2, CD2AP, panAKT, p-AKT (Thr308), PDK1 and p-PDK1 (Ser241) are shown in Supplemental Fig. S2.

### Lentiviral infection

CD2AP was overexpressed in podocytes by lentiviral infection. Untagged human CD2AP cDNA (OriGene Technologies, Rockville, MD, USA) was cloned into pSIN18.cppt.hEF1_p.WPRE vector^44^. Empty pSIN18.cppt.hEF1_p.WPRE vector was used as control. Virus production and infections were performed as previously described^11^.

### SHIP2 activity measurement

CD2AP−/− and wild type podocytes treated with 10 µM AS1949490 for 24 h when indicated were lysed with 1% NP-40, 20 mM Tris-HCl pH 7.5, 150 mM NaCl supplemented with 10 µM AS1949490. SHIP2 was immunoprecipitated from 500–700 µg of lysate with 5 µg of SHIP2 I-20 IgG (Santa Cruz Biotechnology) or 5 µg of purified goat IgG (Invitrogen) as a control. Protein complexes were harvested using protein G sepharose beads (Invitrogen). Beads were washed three times with malachite green assay buffer (10 mM HEPES pH 7.5, 250 mM sucrose, 6 mM MgCl_2_, 0,25 mM EDTA, 0,1% CHAPS) supplemented with 10 µM AS1949490. The phosphatase activity of SHIP2 was measured in malachite green assay buffer containing 100 µM PI(3,4,5)P_3_ (Echelon Biosciences, Salt Lake City, UT), 10 µM AS1949490 and 5 µl of immunoreaction beads to initiate the breakdown of PI(3,4,5)P_3_ to PI(3,4)P_2_, with 50 µl total reaction volume. Samples were incubated at RT for 20 min. 100 µl of Biomol Green (Enzo Life Sciences, Farmingdale, NY) was added followed by 25 min incubation at RT to stop the enzymatic reaction. Absorbance at 630 nm was measured using Fluoroskan Ascent FL (Thermo Fisher Scientific) to detect free phosphate concentration.

### In-Cell Western

In-Cell Western was performed as previously described^18^. Shortly, after permeabilization cells were incubated with goat anti-SHIP2 I-20, rabbit anti-phospho-AKT (Thr308), mouse anti-phospho-AKT (S473), rabbit anti-Pan AKT, anti-phospho-p44/p42 MAPK (T202/Y204) or anti-p44/p42 MAPK IgGs at RT for 1 h, followed by IRDye 800 donkey anti-goat IgG (LI-COR) and 1 µM DRAQ5^TM^ (Thermo Fisher Scientific) at RT for 1 h. Detection and quantification was performed with an Odyssey Infrared Imager (LI-COR). The signal for each protein was normalized with DRAQ5^TM^.

### Immunohistochemistry

Generation of the CD2AP−/− knockout mouse model has been described earlier^5^. Kidney samples of 3 weeks old wild type and CD2AP−/− mice were embedded in Tissue-Tek^®^ O.C.T. Compound (Sakura, Leiden the Netherlands) and fixed with 2% paraformaldehyde (PFA) after sectioning. Sections were stained with anti-8-OHdG (15A3) with Mouse on Mouse Polymer IHC Kit (Abcam, Cambridge, UK). Slides were counterstained with haematoxylin and digitally scanned with 3DHistech Pannoramic 250 FLASH II (3DHISTECH Ltd., Budapest, Hungary). Quantification of the signal in glomeruli (n = 50–100 glomeruli per kidney) of three wild type and four CD2AP−/− mouse kidneys was performed with HistoQuant module (3DHISTECH Ltd.).

### Induction of nephrosis

PA-nephrosis was induced to young male Sprague-Dawley rats weighing 250–280 g by a single intraperitoneal injection of PA (15 mg/100 g, Sigma-Aldrich) in 0.9% saline. Control rats were injected with 0.9% saline. Animals had access to standard chow and water ad libitum. Urine was collected in metabolic cages 0, 3, 6 and 9 days after PA/saline injections. Level of albuminuria was measured by nephelometry (Behring Nephelometer 100 analyzer, Behringwerke AG, Marburg, Germany). The nephelometry results are published earlier^25^. Animals treated with PA or saline were sacrificed 10 days after PA injection (n = 3 per group). The National Animal Experiment Board approved the protocols, and all animal experiments were performed according to the approved guidelines.

### Immunofluorescence microscopy

Rat kidneys were snap-frozen and embedded in Tissue-Tek^®^ O.C.T. Compound (Sakura). Cryosections were fixed with 3.5% PFA, permeabilized with 0.1% Triton X-100 in phosphate-buffered saline (PBS) at RT for 15 min and blocked with CAS-block (Invitrogen). Sections were incubated with rabbit anti-CD2AP (Santa Cruz Biotechnology), quinea pig anti-nephrin IgGs (Sigma Aldrich) and goat anti-SHIP2 I-20 IgGs (Santa Cruz Biotechnology) overnight at +4 °C in ChemMate (Dako Cytomation, Glostrup, Denmark), followed by Alexa Fluor 555 donkey anti-rabbit, Alexa Fluor 488 donkey anti-quinea pig and Alexa Fluor 555 donkey anti-goat IgGs (Invitrogen) together with Hoechst (Fluka, Sigma Aldrich) for 1 h in ChemMate (Dako Cytomation). Samples were mounted in Mowiol and examined with Leica SP2 confocal microscope (Leica Microsystems CMS GmbH, Mannheim, Germany).

### Statistical methods

All cell culture experiments were carried out at least three times. All variables were presented as mean ± SD or ±SEM. The significance of differences between groups was determined by ANOVA or Students *t*-test (GraphPadPrism6, GraphPad Software Inc, La Jolla, CA, USA), and p-values of less than 0.05 were considered statistically significant.

### Data availability

No datasets were generated or analysed in the current study.

## Electronic supplementary material

Supplementary material
