# Supplementary material for: Inhibition of SHIP2 in CD2AP-deficient podocytes ameliorates reactive oxygen species generation but aggravates apoptosis
Source: Sci Rep. 2017 Sep 6;7:10731. doi: 10.1038/s41598-017-10512-w (PMC5587593; doi:10.1038/s41598-017-10512-w)
Supplement: Supplementary file 1 — Supplementary material [file 41598_2017_10512_MOESM1_ESM.pdf]

SUPPLEMENTAL MATERIAL

Inhibition of SHIP2 in CD2AP-deficient podocytes ameliorates reactive oxygen species generation but aggravates apoptosis

Pauliina Saurus, Tuomas A. Tolvanen, Sonja Lindfors, Sara Kuusela, Harry Holthöfer, Eero Lehtonen and Sanna Lehtonen

Supplemental Figure S1. Full width Western blots of cropped blots in Figure 1D and Figure 2D.

(A) Full width blots of p-AKT (Thr308) and panAKT shown in Figure 1D. **(B)** Full width blot of SHIP2 immunoprecipitation shown in Figure 2D. Areas shown in Figures 1D and 2D are marked.

Supplemental Figure S2. Full-length lanes of Western blots using antibodies against SHIP2, CD2AP, AKT, p-AKT (Thr308), PDK1 and p-PDK1 (Ser241).

(A) Western blots of wild-type mouse podocyte and differentiated human podocyte lysates with polyclonal goat-anti-SHIP2 I-20 antibody (Santa Cruz Biotechnology) detected with Alexa 680 donkey-anti-goat antibody, and polyclonal rabbit-anti-CD2AP antibody (homemade, characterized in Lehtonen, S. *et al.*, *J. Biol. Chem.* 275, 32888-32893, 2000) detected with IRDye 800 donkey-anti-rabbit antibody, probed from the same membrane. **(B)** Western blots of wild-type mouse podocyte and differentiated human podocyte lysates with monoclonal mouse-anti-panAKT antibody (R&D Systems) detected with Alexa 680 donkey-anti-mouse antibody, and polyclonal rabbit-anti-phospho-AKT (Thr308) antibody (Cell Signaling Technology) detected with IRDye 800 donkey-anti-rabbit antibody, probed from the same membrane. **(C)** Western blot of wild-type mouse podocyte and differentiated human podocyte lysates with polyclonal rabbit-anti-PDK1 antibody (Cell Signaling Technology) detected with Alexa 680 donkey-anti-rabbit antibody. **(D)** Western blot of wild-type mouse podocyte and differentiated human podocyte lysates with polyclonal rabbit-anti-phospho-PDK1 (Ser241) antibody (Cell Signaling Technology) detected with Alexa 680 donkey-anti-rabbit antibody.

A

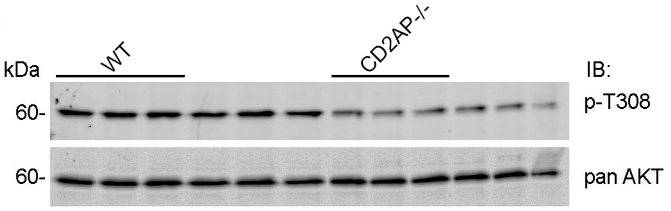

B

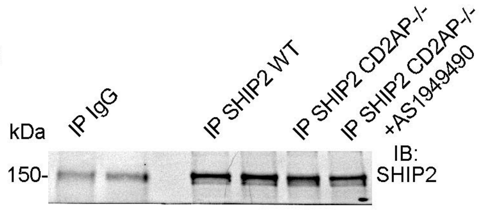

Supplemental Figure S1

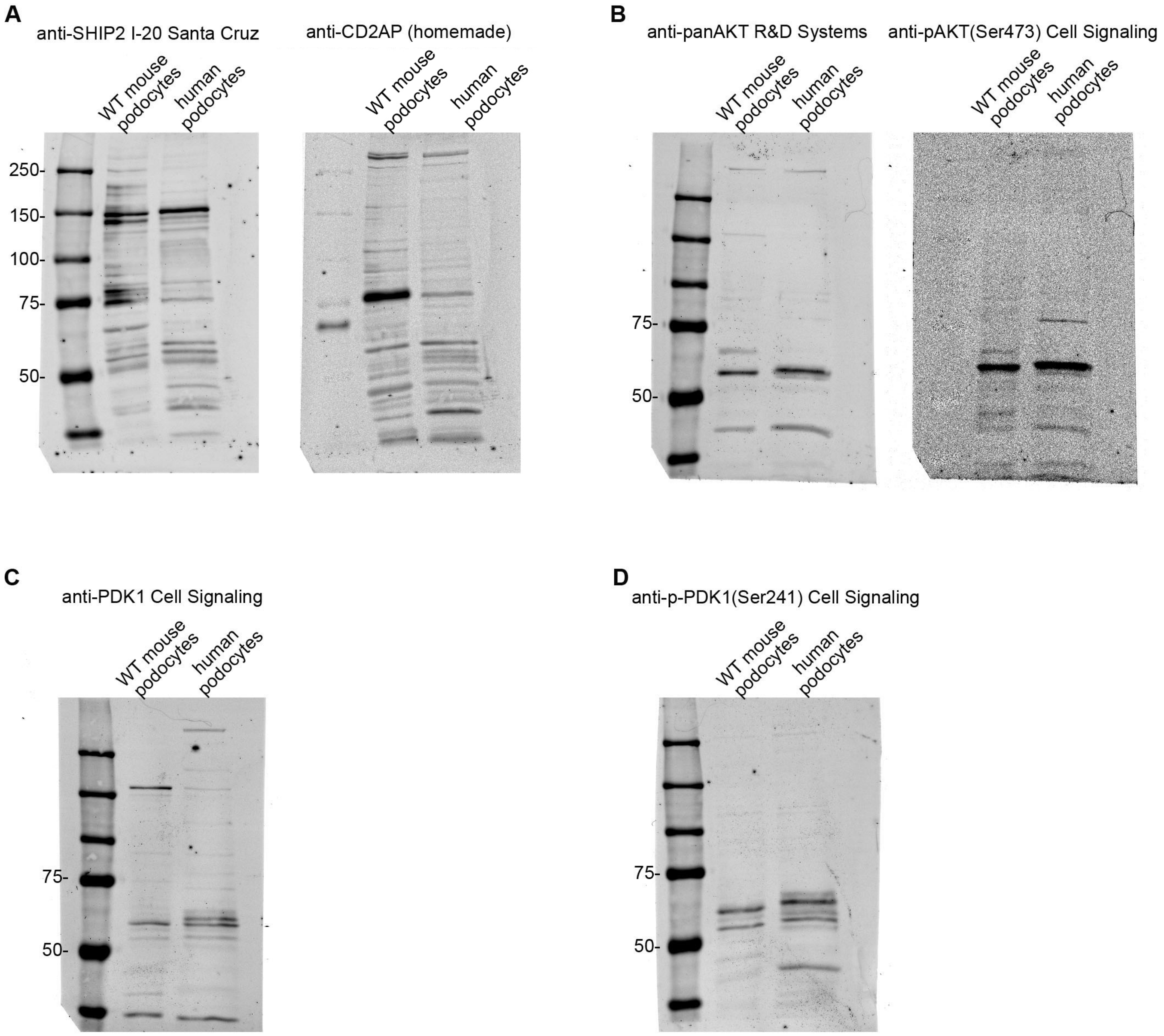

Supplemental Figure S2
